# Inhibitory Effects of Aqueous and Hydroalcoholic Extracts from Jatobá Coat (*Hymenaea courbaril* L.) on Pancreatic Amylase and Starch Absorption

**DOI:** 10.3390/plants14071133

**Published:** 2025-04-05

**Authors:** Ana Caroline Polo, Thaís Marques Uber, Gustavo Henrique Souza, Rúbia Carvalho Gomes Corrêa, José Rivaldo dos Santos Filho, Anacharis Babeto de Sá-Nakanishi, Flávio Augusto Vicente Seixas, Adelar Bracht, Rosane Marina Peralta

**Affiliations:** 1 Postgraduate Program in Food Science, State University of Maringá, Maringa 87020-900, PR, Brazilabsnakanishi@uem.br (A.B.d.S.-N.); 2 Postgraduate Program in Biochemistry, State University of Maringá, Maringa 87020-900, PR, Brazilpg403653@uem.br (G.H.S.); joserivaldosf01@gmail.com (J.R.d.S.F.);; 3 Postgraduate Program in Clean Technologies, Cesumar University—UNICESUMAR, Maringa 87050-390, PR, Brazil; 4 The Mountain Center of Investigation (CIMO), Polytechnic Institute of Bragança, Santa Apolónia Campus, 5300-253 Bragança, Portugal; 5 Associated Laboratory for Sustainability and Technology in Mountain Regions (SusTEC), Polytechnic Institute of Bragança, Santa Apolónia Campus, 5300-253 Bragança, Portugal; 6 Department of Technology and Post-Graduate Program of Molecular and Cell Biology, State University of Maringá, Maringá 87020-900, PR, Brazil; favseixas@uem.br

**Keywords:** diabetes, α-amylase inhibition, starch absorption, glycemia, tannins

## Abstract

Jatobá (*Hymenaea courbaril*) is a native tree abundant in Brazil. The fruit coat is an industrial by-product of jatobá flour processing, typically discarded. Presently, within the circular bioeconomy concept, there are efforts underway that aim at finding economically viable applications for the bio-residues of jatobá. Within this context, the present work attempts to find possible applications for the jatobá coat in glycemic control through inhibition of α-amylase activity. Aqueous and hydroethanolic extracts were used. In vitro experiments included detailed kinetic studies with an α-amylase catalyzed reaction. Starch absorption in vivo was assessed by means of a starch tolerance test in mice. Both extracts inhibited α-amylase. The IC_50_ values for the aqueous and hydroalcoholic extracts were 81.98 ± 3.53 µg/mL and 51.06 ± 0.42 µg/mL, respectively. The inhibition was of the non-competitive type. Both extracts reduced hyperglycemia caused by starch administration in mice, the aqueous extract being effective over a larger dose range. This action can be attributed to the α-amylase inhibition. In silico studies suggested that procyanidin dimers, taxifolin 7-O-rhamnoside, and quercetin 7-rhamnoside contribute, but several other not-yet-identified substances may be involved. The findings suggest that aqueous and hydroalcoholic extracts from jatobá coat warrant further investigations as potential modulators of glycemia following starch ingestion.

## 1. Introduction

Jatobá *(Hymenaea courbaril* L.) is a leguminous tree which occurs abundantly in Brazilian forests. Its economic perspectives are promising not only for its lumber-derived products (wood, resins, incense, cosmetics), but also for its health-beneficial by-products (food ingredients, tonics, fortifiers, and energizing substances) [1,2]. It is vox populi that the plant also possesses medicinal properties such as antimicrobial, antioxidant, anti-inflammatory, antiplasmodic properties, as well as larvicidal activities [1,3]. Extracts from various anatomical parts of this species exhibit significant levels of terpenes and terpenoids, as well as flavonoids, amino acids, fatty acids, carbohydrates, coumarins, steroids, various lactones, and chromones [4]. The extraction of polyphenolic compounds from jatobá (*Hymenaea courbaril* L. var *stilbocarpa*) bark using supercritical fluid with CO_2_ and co-solvents has already been investigated, and it revealed compounds such as malic acid, fructose, quinic acid, sucrose, catechin, and epicatechin [5]. More recently, solid state fermentation using *Aspergillus niger* was used to extract antioxidant phenolics from the jatobá bark [6]. In a study conducted in 2024 [7], three extraction methods, conventional agitated extraction (CAE), supercritical fluid extraction (SFE), and ultrasound-assisted extraction (UAE), were compared and analyzed regarding the economic feasibility of exploiting the bioactives of the jatobá bark on a large scale. The conclusion was that the three methods are economically viable for the recovery of phenolic extracts from jatobá bark, with payback times of 4.2, 2.0, and 3.4 years, respectively. This work shows the economic feasibility and potential scalability of these extraction processes, establishing a basis for future industrial implementation and commercialization of jatobá bark extracts.

**Figure 1:**
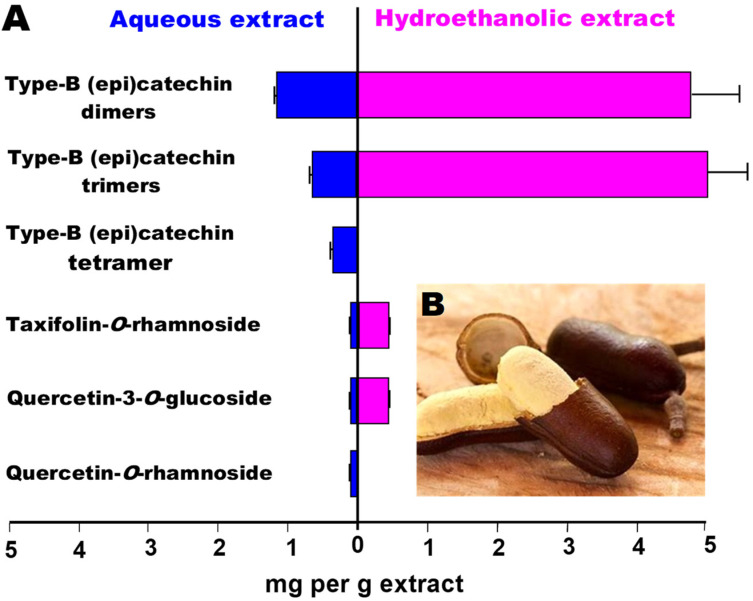
(**A**) Tannins and other polyphenolics profiles of aqueous (**left** panel) and hydroalcoholic (**right** panel) extracts of jatobá coat. For more information, see Del Angelo et al. [14]. (**B**) Pulp and coat of jatobá (*Hymenaea courbaril* L.).

The fruit of jatobá, illustrated in panel B of Figure 1, is a hard-shelled pod that contains a sweet, farinaceous pulp used as a food ingredient known as jatobá flour. The pod coat is an industrial byproduct of flour processing, typically discarded. The fruit coats contain compounds belonging to a group that has been claimed to possess many applications as a biological agent, namely tannins (procyanidin oligomers) of varying degrees of polymerization [8].

Presently, within the circular bioeconomy concept [9], there are series of efforts underway with the purpose of finding economically viable applications for the bio-residues of many plants including Jatobá [10,11,12]. Within this context, the present work shows attempts at finding a possible application for the jatobá coat in the area of glycemic control through inhibition of α-amylase activity. This could be an important application for the jatobá residues, as diabetes is a widely distributed disease that has far-reaching consequences for the quality of human life [13].

Type 2 diabetes in particular is a chronic metabolic disorder characterized by insulin resistance and relative insulin deficiency, leading to elevated blood glucose levels. It is often associated with lifestyle factors such as obesity, physical inactivity, and poor dietary habits [13,15,16,17]. Inhibition of α-amylase is a property that contributes to reduce blood glucose especially after starch rich meals because the absorption of the glucosyl moieties derived from this polysaccharide is substantially retarded [18,19]. Periodic peaks in blood glucose concentration are undesired, especially for individuals with diabetes. It is amply believed that such peaks can be at least attenuated when the activity of the pancreatic α-amylase is diminished up to a certain degree. Some studies suggest that natural α-amylase inhibitors may improve insulin sensitivity, enabling the body to utilize glucose more effectively. This can be particularly beneficial for individuals with insulin resistance, a common feature of type 2 diabetes. Recent research on different edible plants and their wastes such as coats, barks, and seeds rich in phenolic compounds has revealed inhibitory action on key enzymes of carbohydrate digestion, mainly pancreatic α-amylase. These compounds act by binding to the active site of α-amylase, reducing its catalytic activity and slowing down carbohydrate digestion [20,21,22,23]. Inhibition of the α-amylase by jatobá coat extracts is a strong possibility if one considers the richness of the jatobá coat in procyanidin oligomers (tannins). Tannins generally have the capacity to inhibit digestive enzymes, as extensively demonstrated in various studies [11,12,19,24]. An advanced characterization of the phenolic constitution of aqueous and hydroalcoholic extracts of this byproduct has been reported more recently, as illustrated by Figure 1 [14].

Considering, thus, what was exposed above, the objectives of this study were to evaluate, by means of both in vitro and in vivo experiments, the possible inhibitory effects of aqueous and hydroalcoholic extracts of the jatobá bark on the pancreatic α-amylase and starch absorption. Detailed kinetic studies were conducted, with initial reaction rate measurements of the α-amylase catalyzed reaction at various concentrations of substrate (starch) and extracts. The effects on starch absorption in vivo were assessed by means of a starch tolerance test in mice. Complementation of in vitro experiments with in vivo experiments are hoped to provide a more complete picture of the anti-diabetic potential of jatobá bark extract.

## 2. Materials and Methods

### 2.1. Plant Material

The mature pods of *Hymenaea courbaril* were collected at the Iguatemi Experimental Farm of the State University of Maringá, district of Iguatemi (23°25′ S; 51°57′ W; 550 m altitude), Parana State, Brazil. They correspond to those of the voucher specimen with the code HUEM000020252 that was previously deposited at the Herbarium of the State University of Maringá (*Herbanário da Universidade Estadual de Maringá*—HUEM). The pods were collected in August 2023 and handled as described previously [14].

### 2.2. Extraction Procedure

The extracts, aqueous (AE) and hydroethanolic (ETOH), were obtained according to the methodology described previously [14]. These solvents were found to present the highest yields in terms of antioxidant activity [14]. The extraction was performed after separating fragmenting and grounding the pods to a fine powder. Deionized water and an ethanol:water mixture (70:30, *v*/*v*) were used. The proportion of pod residue/extractor solution was established at 1:20 (i.e., 20 mL extractor per g residue). The mixtures were added to vials, which were sealed and shaken, sheltered from light, for 2 h at 130 rpm at room temperature (25 °C). The procedure was repeated three times, and the combined extracts were centrifuged at 1800× *g* for 15 min. Ethanol for removed from the supernatant of the hydroethanolic extract by evaporation at 35 °C. The dry extracts, obtained by lyophilization, were stored at −20 °C before use in the experiments.

### 2.3. In Vitro Assay of Pancreatic α-Amylase

The assays of the porcine pancreatic α-amylase were carried out at 37 °C in 20 mmol/L phosphate buffer, pH 6.9, containing 6.7 mmol/L NaCl [25]. The enzyme and one of the two extracts (aqueous and hydroethanolic) were pre-incubated with the enzyme and the reaction was initiated by adding the substrate, potato starch. The specific activity of the porcine pancreatic α-amylase was 500 units/mg protein. The amount of enzyme added to each reaction system was 1 unit, and the reaction was allowed to proceed for 10 min. The reducing sugars resulting from the hydrolysis of starch were assayed by means of the 3,5 dinitro-salicylic acid (DNS) method, using maltose as standard [26].

### 2.4. In Vivo Experiments

The actions of the jatobá extracts on starch absorption were investigated using the traditional starch tolerance test in male Swiss mice weighing 30–35 g. Handling of the animals obeyed the universal principles of animal care and welfare in experimentation, and the protocols were approved previously by the Ethics Committee on Animal Experimentation at the University of Maringá (Protocol No. 9006100823-CEUA-UEM). The protocols were essentially those described in previous investigations [27,28,29], but were adapted to the particular characteristics of the jatobá extracts in terms of doses and number of experimental animals for each group. Male healthy mice weighing 30–35 g were used in all experiments. The mice were housed, fed, and treated in accordance with the universally accepted guidelines for animal experimentation. Prior to the investigations, the animals were kept for one week under the standard environmental conditions. Throughout the experimentation period, the mice were maintained in single cages and had access to standard pellet diet and water ad libitum. Food was withdrawn 18 h before the experiments. Nine groups of animals were used. To group 1 (positive controls), commercial corn starch (1 g per kg body weight) was administered intragastrically. Group 2 (negative control) received only tap water. Group 3 received intragastrically commercial corn starch plus acarbose (50 mg/kg). Groups 4, 5, and 6 received intragastrically commercial corn starch plus three different doses of the aqueous extract from the jatobá coat (200 mg/kg, 400 mg/kg, and 600 mg/kg, respectively). Groups 7, 8, and 9 received intragastrically commercial corn starch plus three different doses of the hydroethanolic extract from the jatobá coat (200 mg/kg, 400 mg/kg, and 600 mg/kg, respectively). The number of mice effectively used in each experimental series is given in the legends of the graphs showing the results of the experiments. Fasting blood glucose levels were determined before the administration of starch and amylase inhibitors (0 time). Later evaluations of blood glucose levels took place at 15, 30, 45, and 60 min.

Blood glucose from cut tail tips was determined using Accu-Chek^®^ Active Glucose Meter. The areas between the starch tolerance curves and the basal line curves (established after water administration) were computed using MicroMath Scientific Software version 2.0 (Salt Lake City, UT, USA). The ID_50_ values of the relationship between these areas and the extract doses that were administered were computed using Stineman’s interpolation formula using the options available at the MicroMath Scientific Software (Salt Lake City, UT, USA).

### 2.5. Binding Simulations (Docking)

The structure of the pancreatic human α-amylase, crystallized in the presence of the ligand myricetin and the cofactors Ca^2+^ and Cl^−^ (PDBid: 4gqr), obtained at a 1.20 Å resolution, was utilized in the simulations [30]. The crystallographic structure of human pancreatic α-amylase obtained in the presence of the ligand myricetin, and cofactors represents a bound form of the enzyme, in which its active site presents the appropriate conformation for interacting with other ligands. In this case, the presence of the ligand in the catalytic can be used as a structural guide for selecting the best pose of other compounds. Myricetin seems to be a suitable reference because one expects binding of the polyphenolics to be present in the *Hymenaea courbaril* extracts to the α-amylase. Initially, the structure was minimized by means of the NAMD3/VMD program package [31,32]. For accomplishing this, the Charmm36 shape field was used for the protein structure, water molecules, ions, and cofactors, whereas the force field for the ligand myricetin was generated using the server SwissParam in the same format [33]. The system, with all its structural water molecules, was immersed in a periodical box with water TIP3, 150 mM in NaCl, and counter ions of sodium numerous enough for neutralizing the charges. In the first step of minimization, the structure of the ligand myricetin was fixed in space in order to preserve the experimental information. The water molecules, salts, and proteins were minimized by 20,000 steps of Conjugated Gradient (CG). In the second step, the atoms of the ligand were liberated for moving themselves and the system was again minimized by 5000 steps of CG. The resulting structure was utilized in simulations of virtual screening.

For the simulations of virtual screening, the minimized structure was freed of its water molecules and salts, except for the cofactors Ca^2+^ and Cl^−^. The docking program and protocol were chosen by redocking the ligand myricetin. The Vina program [34], implemented using the graphical interface Pyrx [35], utilized a standard search and ranking procedure where the search box with the dimensions of 15, 15, and 20 in its *x*, *y*, and *z* axes, was centralized around the structure of the ligand myricetin.

The ligand library was assembled based on the phenolic compounds found by Del Angelo et al. [14] in the aqueous and hydroethanolic extracts of jatobá (*Hymenaea courbaril*). The structures of the compounds were obtained at the PubCem database (https://.pubchem.ncbi.nlm.nih.gov, accessed on 10 December 2024) based on their respective molecular masses and on the *m*/*z* ratios of the main ions and product ions obtained in the electron spray ionization mass spectrometry experiments conducted by Del Angelo et al. [14], when available. Furthermore, there was also made use of the water to octanol partition coefficient (log P) of each compound in order to correlate it with the corresponding retention time given by Del Angelo et al. [14].

Four virtual screening simulations using the validated protocol of the Vina program were conducted with the whole library plus the reference myricetin (PubChem CID 5281672), totalizing 14 compounds. The selection criteria favored the compounds with scores greater than that of the reference substance and those that presented a pattern of interaction of the drug-like type, where all the poses in all of the four simulations had a root mean square deviation of atomic positions (rmsd) smaller than 1.0 Å.

### 2.6. Curve Fitting and Statistical Analysis

Numerical interpolation for the determination of the half-maximal inhibitor concentration (IC_50_) was performed using the Scientist^®^ software version 2.0 from MicroMath Scientific Software (Salt Lake City, UT, USA). The same program was used for fitting the rate equations to the experimental initial rates of the α-amylase activity by means of an iterative non-linear least-squares procedure. The model selection criterion (MSC), the standard deviations of the optimized parameters, and the sum of squared deviations between experimental reaction rates and calculated ones was used as a guide for deciding about the kinetic mechanism with the highest probability. The model selection criterion is defined as follows [36]:

(1) $$\mathrm{MSC}\;=\;\ln\left[\frac{\sum_{i=1}^{n}w_{i}{\left(Y_{{\mathrm{obs}}_{i}}-{\bar{Y}}_{\mathrm{obs}}\right)}^{2}}{\sum_{i=1}^{n}w_{i}{\left(Y_{{\mathrm{obs}}_{i}}-Y_{\mathrm{cal}}\right)}^{2}}\right]+\frac{2p}{n}$$

Y_obs_ is the measured reaction rates, ${\bar{Y}}_{\mathrm{obs}}$ is the mean reaction rate, Y_cal_ is the reaction rate calculated with the optimized parameters, w is the weight of each experimental point, n is the number of observations, and p is the number of parameters of the set of equations.

For comparing the time evolution of the blood glucose concentrations after the administration of the various jatobá extracts (starch tolerance curves), the data were submitted to multiple variance analysis (MANOVA) with post hoc testing according to Student–Newman–Keuls. Computations were performed using the Statistica^TM^ program for Windows^®^ 95/98/NT from Stat Soft^®^ (Tulsa, OK, USA). The general criterium of significance was *p* ≤ 0.05).

## 3. Results and Discussion

### 3.1. α-Amylase Activity as a Function of the Jatobá Extracts Concentrations

**Figure 2:**
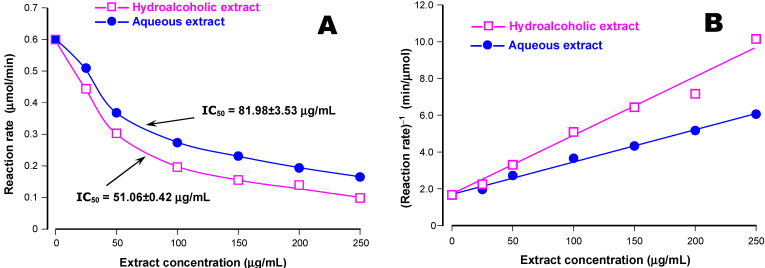
Inhibition of the pancreatic α-amylase by the aqueous and hydroalcoholic extracts of jatobá. Initial rates of the reaction catalyzed by the pancreatic α-amylase were measured as described in the Section 2 at a fixed substrate (starch) concentration (1 g/100 mL) and varying extract concentrations. In panel (**A**), the reaction rates are represented against the extract concentrations; in panel (**B**), the reciprocals of the reaction rates are plotted versus the extract concentrations. The IC_50_ values, given in panel (**A**), were computed by numerical interpolation (Stineman’s interpolation formula).

The results of the experiments in which the influence of the hydroalcoholic and aqueous extracts of the jatobá coat on the α-amylase activity are shown in Figure 2. These experiments were run with a fixed substrate (starch) concentration (1 g/100 mL) and varying concentrations of both extracts. In panel A of Figure 2, the reaction rates were represented against the extracts concentrations. Both extracts were inhibitory, but the hydroalcoholic one was more effective. The IC_50_ values, which are indicated on Figure 1A, can be used to quantify the difference in effectiveness of both extracts, which is around 60% in favor of the hydroalcoholic extract.

The representation of the reciprocal reaction rates (1/v) against the concentrations of both extracts in Figure 2B was performed in order to obtain a preliminary answer about the mechanism of the inhibition, i.e., whether it is incomplete (hyperbolic) or complete (linear or parabolic). The rather linear relationships between 1/v and the extracts concentrations suggest that the inhibition is of the linear type [37].

### 3.2. Inhibition of α-Amylase Activity at Various Substrate Concentrations

**Figure 3:**
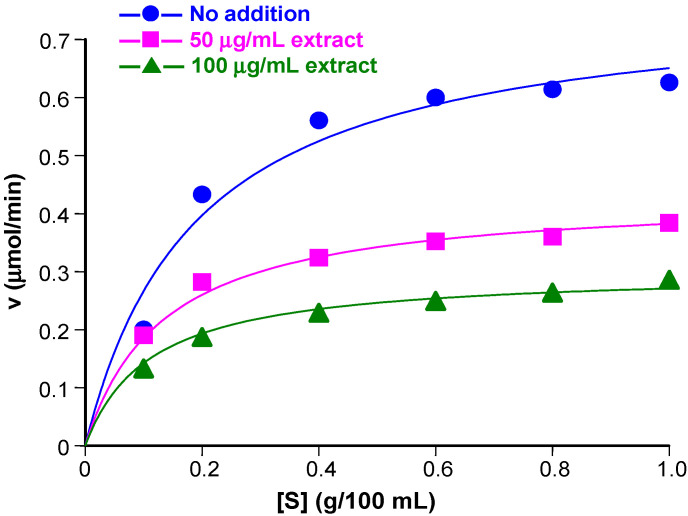
Kinetics of the inhibition of the pancreatic α-amylase by the aqueous extract of jatobá. Initial rates of the reaction catalyzed by the pancreatic α-amylase were measured as described in the Section 2 at various substrate concentrations ([S], starch) and the aqueous jatobá extract (indicated at the right top). Equation (2) was fitted simultaneously to the whole dataset by means of a non-linear least-squares procedure. The symbols represent the experimental data (n = 2). The continuous lines were calculated using Equation (2) after introducing the optimized kinetic constants. The correlation coefficient (r) was 0.993 and the model selection criterium was 4.032.

**Figure 4:**
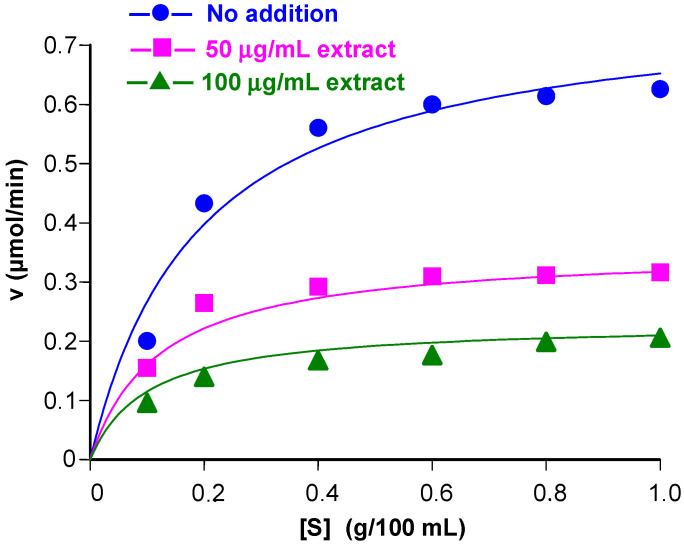
Kinetics of the inhibition of the pancreatic α-amylase by the hydroethanolic extract of jatobá. Initial rates of the reaction catalyzed by the pancreatic α-amylase were measured as described in the Section 2 at various substrate concentration ([S], starch) and the hydroalcoholic jatobá extract (indicated at the right top). Equation (2) was fitted simultaneously to the whole dataset by means of a non-linear least-squares procedure. The symbols represent the experimental data (n = 2). The continuous lines were calculated using Equation (2) after introducing the optimized kinetic constants. The correlation coefficient (r) was 0.992 and the model selection criterium was 3.822.

To extend the analysis further, initial reaction rates were measured by simultaneously varying the substrate and the extracts concentrations. The results of these experiments are shown in Figure 3 and Figure 4. In these graphs, the initial rates are represented against the substrate concentrations. For each inhibitor, the rates at varying substrate concentrations were measured with two different inhibitor concentrations in addition to the condition of inhibitor absence (control curves). The inhibitor concentrations are indicated in each graph. Typical saturation curves were obtained, confirming previous observations [29,38]. For both extracts, there is no tendency of convergence at high substrate concentrations, a fact that excludes competitive inhibition. Consistently, the equation for competitive inhibition did not fit to the experimental data. Attempts at fitting the equation for uncompetitive inhibition also failed, but equation for non-competitive inhibition [37,39], given below, fitted quite well to the experimental data:

(2) $$v=\frac{V_{\max}[S]}{K_{M}\left(1+\frac{\left[I\right]}{K_{i1}}\right)+[S]\left(1+\frac{\left[I\right]}{K_{i2}}\right)}$$

In Equation (2), v is the reaction rate, [S] is the substrate concentration, and [I] is the extract concentration. Additionally, V_max_ is the maximal reaction rate, K_M_ is the Michaelis-Menten constant, K_i1_ is the dissociation constant (inhibitor constant) of the enzyme-inhibitor complex (EI), and K_i2_ is the dissociation constant (inhibitor constant) of the enzyme–substrate–inhibitor complex (ESI). The agreement between theory and experiment can be appreciated by comparing the experimental points in Figure 3 and Figure 4 with the continuous lines, which represent the curves that were calculated after introducing the optimized values of V_max_, K_M_, K_i1_, and K_i2_ into Equation (2) for each inhibitor in addition to the values of [S] and [I]. For the aqueous extract the optimized values were as follows: V_max_, 0.774 ± 0.031 μmol/min, K_M_, 0.191 ± 0.026 g/100 mL, K_i1_ = 197.73 ± 147.21 μg/mL, and K_i2_ = 63.55 ± 7.73 μg/mL; for the hydroethanolic extract, optimization produced V_max_, 0.776 ± 0.036 μmol/min, K_M_, 0.190 ± 0.030 g/100 mL, K_i1_ = 128.97 ± 99.81 μg/mL, and K_i2_ = 42.25 ± 5.58 μg/mL. Values of the correlation coefficients and of the model selection criteria (calculated according to Equation (1) are given in the legends. These parameters provide a quantitative measure for the goodness of fit.

Comparison of the inhibitor constants obtained for the aqueous extract (Figure 3) with those obtained for the hydroalcoholic extract (Figure 4) reveals that the latter were smaller, corroborating the fact that the hydroalcoholic extract is a stronger inhibitor when compared to the aqueous extract. For both extracts, K_i2_ is smaller than K_i1_, indicating that, for both preparations, the ESI forms more easily than the EI. In other words, the binding of the substrate to the enzyme facilitates inhibition to a certain degree. Strictly speaking, K_i1_ and K_i2_ are only simple dissociation constants if the inhibitory actions of the extracts are caused in each case by a single substance. This is highly improbable, however, because plant extracts have generally a highly complex molecular composition. For this reason, K_i1_ and K_i2_ are most probably complex composite parameters that reflect the action of several compounds. Since all compounds in the extracts are present in constant proportions, Ki_1_ and K_i2_ can still be regarded as a measure for the inhibitory strength of each preparation [40].

The linear relation between 1/v and [I], shown in Figure 2B, is also in accordance with Equation (2). In fact, if the reciprocal form of Equation (2) is rearranged as a function of [I], it is easy to see that 1/v is a linear function of [I]:

(3) $$\frac{1}{v}=\left(\frac{K_{M}+[S]}{V_{\max}[S]}\right)+\left(\frac{K_{M}}{V_{\max}K_{i1}[S]}+\frac{1}{V_{\max}K_{i2}}\right)\cdot [I]$$

The slope of the 1/v versus [I] line was found to be higher for the hydroalcoholic extract because the K_i1_ and K_i2_ values for this preparation were smaller (Figure 2B). Furthermore, convergence of the straight lines obtained with both extracts in Figure 2B at the 1/v axis is expected at a fixed substrate concentration. It is worth mentioning that linear inhibition indicates that binary complexes, such as EI_2_ or those of higher orders, are not formed, even though it may not be always the same type of molecule that is involved. The formation of complexes such as EI_2_ or ESI_2_ would result in parabolic inhibition instead of the linear inhibition that was effectively observed.

### 3.3. Starch Tolerance Tests in Mice

**Figure 5:**
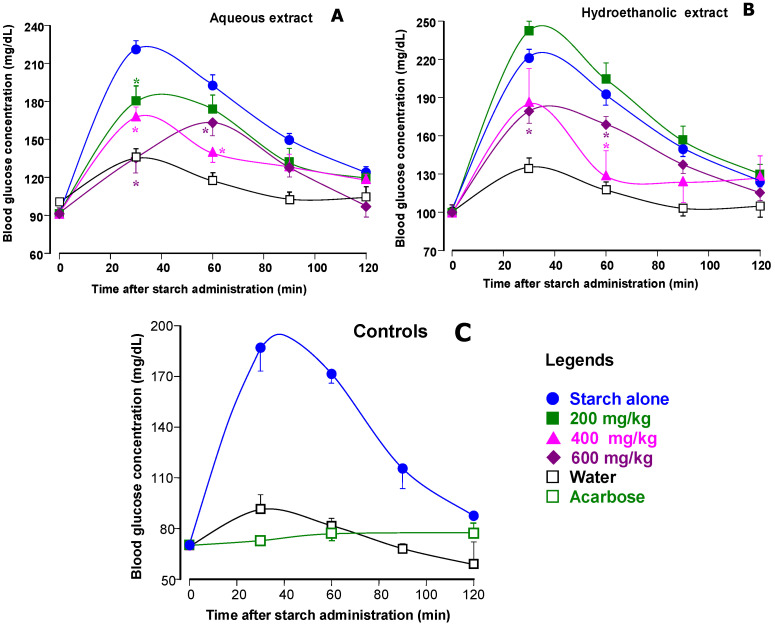
Effects of the aqueous jatobá extract (panel (**A**)), hydroethanolic jatobá extract (panel (**B**)), and acarbose or water (panel (**C**)) on blood glucose levels after oral starch administration in mice. The oral administration of commercial starch (1 g per kg body weight) was performed immediately after the administration of the extracts or acarbose (reference inhibitor). The doses of each extract are given on the graphs. Plasma glucose was measured as described in the Section 2. Each value represents the mean ± mean standard error of a minimum of 5 and a maximum of 22 mice. Asterisks indicate statistical significance relative to the control curve (*p* ≤ 0.05) according to a Student–Newman–Keuls post hoc testing performed after multiple variance analysis (MANOVA).

Inhibition of α-amylase in vivo should, in principle at least, diminish the appearance of free glucose in the circulation after starch ingestion. This phenomenon is frequently estimated by means of starch tolerance tests [24]. Figure 5 shows the results obtained in a series of experiments in which three different doses of both extracts were given to mice before starch administration. Measurements of blood glucose were performed at various subsequent times. Figure 5A and Figure 5B show the results obtained when aqueous and hydroalcoholic extracts were administered, respectively. As expected from numerous previous reports [24,27,41,42], glucose concentration in blood rises rapidly after starch administration under control conditions, but declines thereafter. This response was almost abolished when acarbose, the classical inhibitor of starch digestion, was administered at 50 mg/kg (Figure 5C). Experiments were also performed in which pure water was administered. Ideally, water administration alone should not affect blood glucose. However, manipulation of the animals, even if it does not cause any pain, always produces some stress. Even if the latter is minimized, the consequence will generally be some rise in the blood glucose levels, as shown in Figure 5. The curve herewith obtained is important because it represents the basal line for calculating the true hyperglycemic outburst that occurs in consequence of the absorption of the exogenous glucosyl units derived from starch hydrolysis. Administration of 200 mg/kg of the aqueous jatobá extract was not very effective, as only the peak blood glucose concentration was diminished (Figure 5A). When the doses of the aqueous extract were increased, however, the effects gained significance. The 400 mg/kg dose did not modify the form of the curve, as the peak still occurred at 30 min. The 600 mg/kg dose, however, displaced the peak to 60 min.

The hydroalcoholic extract, as revealed by Figure 5B, showed a more complex behavior, even though the effects were inhibitory at the highest doses. The lowest dose, 200 mg/kg, tended to increase the blood glucose levels, without statistical significance, however. The 400 and 600 mg/kg doses, on the other hand, were solely inhibitory. The time courses of the curves obtained with these two doses were different, however, in that the 400 mg/kg curve presented a sharp drop in glucose concentration initiating at 60 min.

### 3.4. Comparison of the Actions of the Jatobá Extracts with Those of Other Plant Extracts

It is of interest, at this point, to compare the inhibitory strengths of the jatobá extracts with those of other plant extracts. The conditions of the α-amylase assay and the source of the enzyme are important, and, for this reason, only results obtained under very similar conditions should be selected for comparisons. In most studies published so far, only in vitro characterizations of the inhibition of α-amylase and α-amylase are presented, in addition to in silico studies aiming at uncovering mechanistic details of the inhibition [43,44,45]. Although meritorious, such studies do not answer the most important question, which is one related to the in vivo effects. Fortunately, a considerable number of simultaneous in vitro and in vivo approaches have also been published. The most usual in vivo technique is the starch tolerance test, for which Figure 5 provides a good example. A fundamental point of a successful double in vitro–in vivo approach, however, is to conduct the experiments in such a way that numerical parameters can be obtained for comparing the corresponding inhibitory potencies. This can be performed, for example, by comparing IC_50_, the extract concentrations that causes 50% inhibition of the enzymes with the ID_50_, and the in vivo dose that causes 50% diminution of the area between the tolerance curve and the baseline. The baseline is indispensable because the basal glucose levels are high (e.g., 90 mg/dL), and it can vary along the experimental period, as shown in Figure 5 by the experiments in which only water was administered to the mice. The area between the curves in which starch was given to the animals and the basal line obtained when only water is given provides, thus, a measure of the increase in circulating glucose under the specific experimental conditions. It should approach zero when no absorption occurred. Table 1 was constructed with data obtained in the present work and by other investigations found in the literature in which the assays were conducted according to the minimal requirements described above. The list in Table 1 is displayed in decreasing order of potency, as deduced from the corresponding IC_50_ values. The data reveal that the α-amylase inhibitory potency of plant extracts varies by more than two orders of magnitude when the enzyme is assayed in vitro. The jatobá extracts used in the present work are certainly not the most potent inhibitors of the α-amylase, but their potency is close to that of the *Araucaria angustifolia* seed coat extract [28]. Furthermore, the jatobá extracts are much more potent than the purple tea and the jabuticaba peel extracts [29,42].

When the starch tolerance tests are repeated with several doses, it is possible to determine ID_50_. The ID_50_ values for the jatobá extracts listed in Table 1 were obtained from the data shown in Figure 5. After evaluation of the areas between the curves obtained after starch administration and the curves after water administration, the area versus dose curves were analyzed by numerical interpolation for obtaining the ID_50_ values. The curve obtained upon administration of the hydroethanolic extract was biphasic, so that actually two ID_50_ values must be considered. Both are smaller than the ID_50_ value found for the aqueous extract, an observation which is, in this case, consistent with the determined IC_50_ values for the α-amylase inhibition. The ID_50_ values for the data obtained from the literature were either informed directly by the cited authors or calculated by numerical interpolation from the data that were presented. The sole exception is the ID_50_ of the *Araucaria angustifolia* extract, which is not very precise because it was obtained by linear extrapolation. When taken as a whole, the data in Table 1 reveal that there is no correlation between the IC_50_ values for α-amylase inhibition and the ID_50_ values for starch absorption. The phenomenon again corroborates the notion that it is very difficult to predict the in vivo effect on starch digestion based solely on α-amylase or α-glucosidase inhibition measurements [46]. The greatest discrepancy is perhaps the jabuticaba fruit peel extract, which is a very poor inhibitor of α-amylase, but whose effects on starch digestion cannot be classified in the same category. The most efficient inhibitor of α-amylase, the tamarind seed extract, can also be considered a good inhibitor of starch digestion, but somewhat inferior to purple tea, which is also a relatively poor inhibitor of α-amylase. The unexpectedly strong actions of purple tea and of the jabuticaba fruit peel extract is probably related to the fact that diminution of starch digestion by these preparations is not solely caused by inhibition of the α-amylase. Other mechanisms may be co-responsible for the in vivo effects. A question that is more relevant for the present work is one concerned with the relatively modest actions on starch digestion of several extracts that are actually relatively strong inhibitors of α-amylase. This includes, for example, the *Cytinus hypocistis* [24], but also both jatobá extracts. The results of the present work do not allow us to infer about the causes of this discrepancy. In is true, however, that conditions in the gastrointestinal differ quite certainly from those in the incubation system used to assay the α-amylase. Although the analysis that is being conducted here is restricted to the reports in which both IC_50_ and ID_50_ have been determined, a rather qualitative analysis of starch tolerance curves presented in several literature reports, when compared to those listed in Table 1, confirms the absence of correlation between the potency of the in vitro α-amylase inhibition and the capacity of inhibiting starch absorption in vivo. In this respect, it is worth to mention, as examples, the studies performed with extracts obtained from *Cinnamomum verum* (Ceylon cinnamon) [41], *Andrographics paniculata* [47], and *Echinops spinosus* [48].

**Table 1:** Comparison of α-amylase inhibitory strengths of various plant preparations with their capacity in decreasing starch digestion. The IC_50_ values (extract concentrations producing 50% inhibition of α-amylase) were all obtained directly from the cited references. The ID_50_ values are the doses in mice or rats that reduced by 50% the areas under the glucose curves between zero time and 120 min time compared to control experiments. They were either informed directly by the cited authors or calculated from the data that were presented, with the sole exception of *Araucaria angustifolia* data, which are not very precise because they were obtained by linear extrapolation.

Plant Species	Kind of Preparation	α-Amylase Inhibition In Vitro (IC_50_ in µg/mL)	Starch Digestion Inhibition In Vivo (ID_50_ in mg/kg)	Animal	References
*Tamarindus indica* (tamarind)	Seed hydroethanolic extract	13.3	151.4	Mouse	[49]
*Cytinus hypocistis*	Whole plant ultrasound extract	14.0	349.8	Mouse	[24]
*Araucaria angustifolia*	Seed coat extract (rich in tannins)	45.0	≈308.6	Rat	[28]
*Hymenaea courbaril* (jatobá)	Fruit coat hydroalcoholic extract	51.1	385.7 and 436.3 *	Mouse	This work
*Hymenaea courbaril* (jatobá)	Fruit coat aqueous extract	81.9	500.1	Mouse	This work
*Vitis vinifera* (grape)	Grape pomace hydroethanolic extract	145.0	131.6	Rat	[27]
*Camellia sinensis* (purple tea)	Aqueous extract (tea preparation)	630.0	96.8	Mouse	[42]
*Myrciaria jaboticaba* (jabuticaba)	Fruit peel hydroethanolic extract	1963.0	250.0	Mouse	[29]

* The area versus dose curve presented a minimum at the 400 mg/kg dose and, consequently, the 50% inhibition line was intersected twice by the interpolation curve.

It is difficult, thus, to predict the response to each plant extract, especially when the inhibitors are also chemically distinct, because they can be enzymatically modified into either more or less active components. In the case of the *Cytinus hypocistis* extract, attempts were made to answer this question by subjecting the extract to in vitro gastrointestinal digestion [24]. It was found that the latter procedure increased the IC_50_ for α-amylase inhibition by a factor of three. The inhibitory efficiency in the intestinal tract can also be modified by binding to peptides or proteins which are present in the latter [50,51]. In the case of the *Cytinus hypocistis* extract, for example, it was shown that albumin, usually present in the intestinal tract, significantly diminishes its α-amylase inhibitory action in a concentration-dependent manner [24]. Similar phenomena could have happened in the experiments conducted herein with the jatobá extract. Confirmation evidently needs additional experimental work.

It must be stressed that it is equally possible that modifications in the intestinal tract can produce compounds that stimulate rather than inhibit the α-amylase. A balance between inhibition and stimulation may be established under such circumstances. This might indeed have happened in the present work with the hydroethanolic extract when given to mice at the lowest dose as the phenomena in Figure 5 are highly dynamic and time dependent. Plant extracts generally have a highly complex composition and usually differ considerably from each other so that the transformation patterns can also be expected to differ from case to case.

### 3.5. Compounds Possibly Involved

Plant extracts generally have a highly complex composition, a feature that makes it difficult to attribute particular effects to specific compounds. Del Angelo et al. [14], in a recent work, demonstrated the presence of procyanidins and polyphenolics in both the aqueous and hydroethanolic extracts of jatobá, as depicted in Figure 1. It is well known that tannins and polyphenolics, in general, are inhibitors of α-amylase, a fact demonstrated directly by experiments in which purified tannins or polyphenolics were used [38] or by experiments using tannin-rich extracts [24]. The hydroethanolic extract of jatobá was a somewhat more potent inhibitor of α-amylase, an observation that would be consistent with the fact that this preparation has a higher tannin content according to Del Angelo et al. [14] (Figure 1). However, the difference in inhibitory potency (37.7%) between both extracts is less pronounced when compared to their difference in tannin and polyphenolics contents (78%). Consequently, it is possible that procyanidins and polyphenolics are not the sole inhibitors of α-amylase. Nonetheless, it is certainly of interest to find out which of the procyanidins and polyphenolics that were detected in the jatobá extracts are the most active inhibitors of α-amylase. This can be performed by computer simulations (docking simulations), but it requires a more precise identification of the various procyanidins that are present in the extracts. On this respect, the work of Del Angelo et al. [14] needs to be complemented, as these authors did not identify the various epicatechin dimers (3), trimers (3), and the tetramer presumably present in the aqueous and hydroethanolic extracts. This complementary analysis was performed in the present work, and the results are presented in Table 2, in addition to some of the original data of Del Angelo et al. [14]. The latter and the information obtained at the PubChem database (pubchem.ncbi.nlm.nih.gov, accessed on 10 December 2024) were combined to deduce the structure of the various compounds. In Table 2, the compounds were ordered according to their retention times (R_t_) in the high-performance liquid chromatography procedure performed by Del Angelo et al. [14]. Spectroscopic parameters, such as the main ion obtained in the negative mode (*m*/*z* [M–H–] of the electron spray ionization mass spectrometry and the corresponding product ion that was identified by Del Angelo et al. [14], were compared with the description at the PubChem entry. Additionally, physico-chemical parameters such as molecular mass and the water to octanol partition coefficient (log P) were also considered when sorting the most probable compounds. However, in spite of the efforts, for three peaks, namely 2, 7, and 10, it was not possible to identify specifically the compound among two equally possible variants, which were, in these cases, included in the virtual library used for the docking simulations.

**Figure 6:**
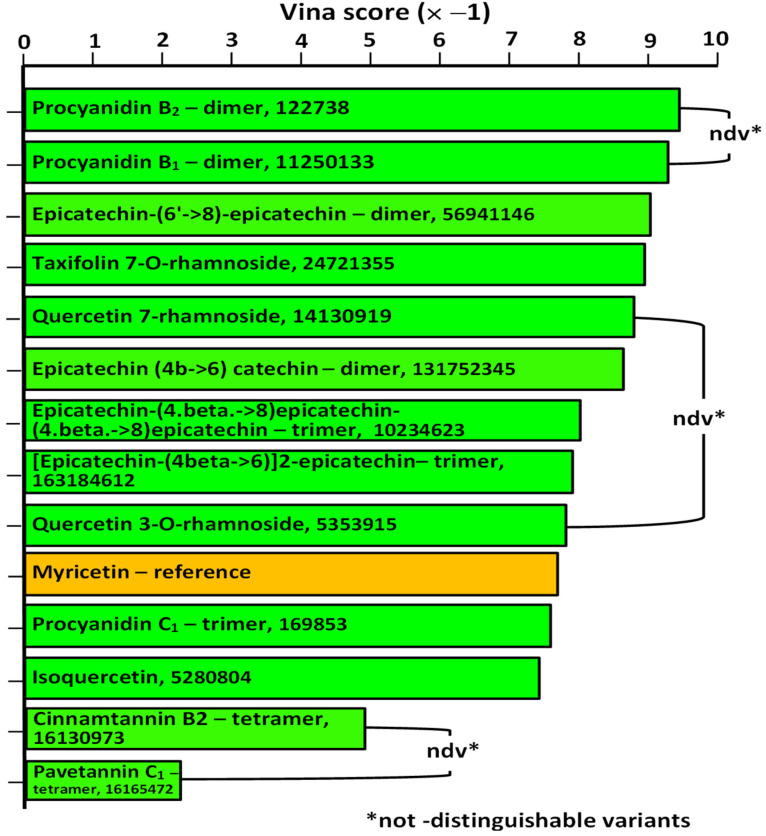
Mean Vina scores obtained when docking the compounds identified in the *H. courbaril* extracts, plus the reference ligand myricetin. The three non-distinguishable variant pairs, numbered in Table 2 as 2*–2*, 7**–7**, and 10***–10***, are indicated by connecting loops. More details about calculations and compound identification are given in the text.

**Figure 7:**
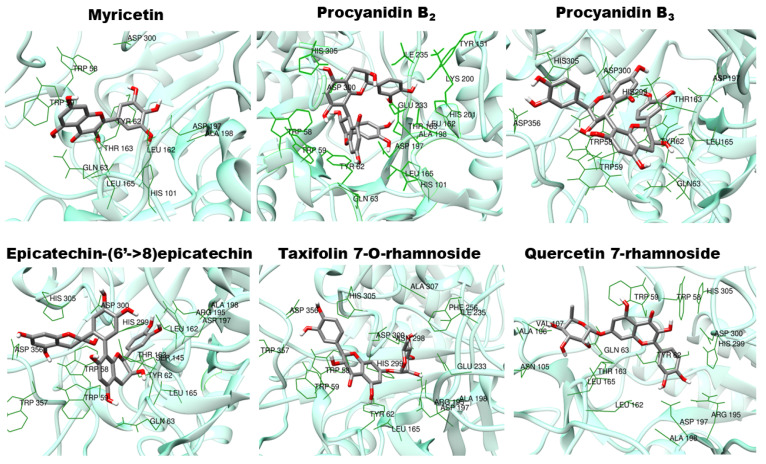
Superposition of the ligands with the highest scores with the pancreatic α-amylase after four simulations. In all the poses shown, the RMSD was under 1.0 Å. All residues in the protein that make contacts of up to 4.0 Å with the corresponding ligand are marked as dark green sticks.

Five docking programs were evaluated by redocking the reference ligand myricetin to the crystallographic complex and also to the minimized complex. Solely, the Vina program was able to yield reproducible results where the poses of the reference ligand presented a root mean square deviations smaller than 1.0 Å. Consequently, the whole virtual screening was conducted with the Vina program. The results are shown in pictorial form in Figure 6, and the numerical mean values are listed in Table 3. From the thirteen compounds of the library, nine presented mean scores higher than that of the reference. However, only the five best ranked presented a drug-like pattern of interaction with the enzyme. These interactions can be seen in Figure 7. In the latter, all residues that make contacts of up to 4.0 Å with the corresponding ligand are marked as dark green sticks. Unfortunately, no indications were found in the registers of the Binding [52] or in the server of the Swiss Target Prediction [53] predictions that any of the nine best ranked compounds in Figure 6 could be able to bind to a glucosidase, including α-amylase. However, the literature reports that catechin, epicatechin, and quercetin are capable of inhibiting the salivary α-amylase [54]. The best ranked compounds in Figure 6 possess these compounds in their core structures, which reinforces the possibility that they could be real inhibitors of the pancreatic α-amylase. Even so, one must seriously consider the presence in the jatobá extracts of other compounds not belonging to the procyanidin or polyphenolics classes. This is a possibility that was explored in the case of the tamarind seed for which several compounds, not belonging to the tannin and polyphenolics classes, but with potential of being strong inhibitors of α-amylase, were detected [49].

## 4. Conclusions

The results obtained in the present work confirm the hypothesis that was raised initially, namely, that thanks to its richness in tannins and polyphenolics, jatobá coat extracts should be able to inhibit α-amylase activity. The hydroethanolic extract was a stronger inhibitor of α-amylase when compared to the aqueous extract (37.7% more active). The aqueous extract, however, produced a much well-defined inhibition of starch digestion in vivo. Compounds such as the procyanidin dimers, taxifolin 7-*O*-rhamnoside, and quercetin 7-rhamnoside are most probably contributing to the α-amylase inhibition, but several other not-yet-identified substances may be involved. Although both extracts bear potential for being used as preparations capable of reducing hyperglycemia caused by starch administration, the aqueous extract seems to be more indicative of an antihyperglycemic agent because it is apparently inhibitory over a more ample range of doses. The weak point of the present study does not differ from the overwhelming majority of similar studies with plant extracts. Since the human health is the ultimate goal of any therapeutic preparation, it is essential to conduct clinical tests in order to confirm or not the applicability of the results that were obtained in animal experiments. There is no doubt, however, that the results obtained herein suggest that both aqueous and hydroalcoholic extracts from jatobá coat warrant further investigations as potential modulators of glycemia following starch ingestion.

## Figures and Tables

**Table 2:** The phenolic compounds detected chromatographically by Del Angelo et al. [14] in the *Hymenaea courbaril* extracts and their possible identification based on the PubChem database.

Data from Del Angelo et al. [14]			Data Obtained at the PubChem Database (https://pubchem.ncbi.nlm.nih.gov, accessed on 10 December 2024)					
UHPLC Peak	R_t_ (min)	Tentative Preliminary Identification by ESI-MS	Compound Name	CID	MW (g/mol)	log P	*m*/*z* Precursor [M-H]-	*m*/*z* Product ion
**1**	4.86	Type-B (epi)catechin dimer	Epicatechin (4b->6) catechin	131752345	578.5	1.5	dna	dna
2 *	5.09	Type-B (epi)catechin dimer	Procyanidin B1	11250133	578.5	2.4	577.14	407.07
2 *	5.09	Type-B (epi)catechin dimer	Procyanidin B2	122738	578.5	2.4	577.14	407.07
**3**	6.17	Type-B (epi)catechin trimer	Procyanidin C1	169853	866.8	3.3	865.23	407.09
**4**	6.95	Type-B (epi)catechin dimer	Epicatechin-(6′->8)-epicatechin	56941146	578.5	2.5	dna	dna
**5**	9.83	Type-B (epi)catechin trimer	[Epicatechin-(4beta->6)]2-epicatechin	163184612	866.8	3.3	dna	dna
**6**	10.49	Type-B (epi)catechin trimer	Epicatechin-(4.beta.->8)epicatechin-(4.beta.->8)epicatechin	10234623	866.8	3.3	865.19	695.10
7 **	11.11	Type-B (epi)catechin tetramer	Cinnamtannin B2	16130973	1153.0	4.3	dna	dna
7 **	11.11	Type-B (epi)catechin tetramer	Pavetannin C1	16165472	1153.0	4.3	dna	dna
**8**	17.55	Taxifolin-*O*-rhamoside	Taxifolin 7-O-rhamnoside	24721355	450.4	0.2	449.11	285.04
**9**	17.91	Quercetin-3-*O*-glucoside	Isoquercetin	5280804	464.4	0.4	463.41	301.25
10 ***	21.34	Quercetin-*O*-rhamnoside	Quercetin 3-*O*-rhamnoside	5353915	448.4	0.9	445.08	301.04
10 ***	21.34	Quercetin-*O*-rhamnoside	Quercetin 7-rhamnoside	14130919	448.4	0.9	447.09	301.03
		Reference	Myricetin	5281672	318.2	1.2	315.01	317.02

*, **, *** Both compounds fit into the description at the PubChem database; dna = data not available; UHPLC = ultra-high-performance liquid chromatography; ESI-MS = electron spray ionization mass spectrometry; CID = PubChem compound identifier; MW = molecular mass; log P = logarithm of the water to octanol partition coefficient; *m*/*z* = mass-to-charge ratio.

**Table 3:** Mean scores obtained from docking simulations with compounds identified in extracts of *H. courbaril*.

Molecule	PubChem CID	Vina Score	
		Mean	SD
Myricetin	5281672	−7.70	0.00
Epicatechin (4b->6) catechin	131752345	−8.63	0.05
Procyanidin B1	11250133	−9.30	0.00
Procyanidin B2	122738	−9.40	0.00
Procyanidin C1	169853	−7.60	0.00
Epicatechin-(6′->8)-epicatechin	56941146	−9.00	0.00
[Epicatechin-(4beta->6)]2-epicatechin	163184612	−7.88	0.72
Epicatechin-(4.beta.->8)epicatechin-(4.beta.->8)epicatechin	10234623	−8.00	0.00
Cinnamtannin B2	16130973	−4.90	0.59
Pavetannin C1	16165472	−2.25	0.29
Taxifolin 7-O-rhamnoside	24721355	−8.93	0.05
Isoquercetin	5280804	−7.43	0.10
Quercetin 3-O-rhamnoside	5353915	−7.80	0.00
Quercetin 7-rhamnoside	14130919	−8.80	0.00

## Data Availability

Data will be made available on request.
